# Synthesis and Isomerization of Some Novel Pyrazolopyrimidine and Pyrazolotriazolopyrimidine Derivatives

**DOI:** 10.3390/molecules19055459

**Published:** 2014-04-25

**Authors:** Aymn E. Rashad, Ahmed H. Shamroukh, Randa E. Abdel-Megeid, Hatem S. Ali

**Affiliations:** 1 Photochemistry Department, National Research Center, Dokki, Cairo 1258943, Egypt; E-Mails: ahshamroukh@yahoo.com (A.H.S.); randaabdelmegeid@yahoo.com (R.E.A.-M.); 2 Chemistry Department, Faculty of Science and Human Studies, Shaqra University, Huraiymla 11961, Kingdom of Saudi Arabia; 3 Chemistry Department, Faculty of Science, Hail University, Hail 81411, Kingdom of Saudi Arabia; 4 Chemistry Department, Faculty of Education, Shaqra University, Afif 11921, Kingdom of Saudi Arabia; 5 Food Science and Nutrition Department, College of Food Science and Agriculture, King Saud University, Riyadh 11564, Kingdom of Saudi Arabia; E-Mail: hali@ksu.edu.com

**Keywords:** pyrazoles, pyrazolopyrimidines, pyrazolotriazolopyrimidines, isomerization

## Abstract

4-Imino-1-*p*-tolyl-1,4-dihydropyrazolo[3,4-*d*]pyrimidin-5-ylamine (**2**) and (1-*p*-tolyl-1*H*-pyrazolo[3,4-*d*]pyrimidin-4-yl)-hydrazine (**3**) were prepared starting from ethyl 4-cyano-1-*p*-tolyl-*1H*-pyrazol-5-ylimidoformate (**1**). The structure of compound **3** was confirmed through preparation of the pyrazole derivatives **4** and **5**. Also, the synthesis and structural characterization of pyrazolo[4,3-*e*][1,2,4]triazolo[4,3-*c*]pyrimidine derivatives **7** and **9** and their isomerization to pyrazolo[4,3-*e*][1,2,4]triazolo[1,5-*c*]pyrimidine derivatives **6** and **8**, respectively, under different suitable reaction conditions were reported. Moreover, the syntheses of 2-substituted-pyrazolo[4,3-*e*][1,2,4]triazolo[1,5-*c*]pyrimidine derivatives **10** and **11** was described.

## 1. Introduction

As part of our ongoing research program on heterocyclic compounds which may serve as leads for designing novel chemotherapeutic agents, we were particularly interested in pyrazoles and fused pyrazolopyrimidines [1,2]. Pyrazolo[3,4-*d*]pyrimidines and their related fused heterocycles are of considerable significance chemical and pharmaceutical utility as purine analogs [3] and many of their derivatives were reported to possess antiviral [1,4] antimicrobial [2,5] anti-inflammatory [6,7] anticancer [8] and xanthine oxidase inhibitor [9] activities.

Moreover, the synthesis of fused triazolopyrimidine moieties has been described by many investigators and these compounds have been proved to have pronounced biological activities [2,10,11,12,13]. Previous observations revealed that the [1,2,4]triazolo[4,3-*c*]pyrimidine derivatives can isomerize under different suitable reaction conditions to the thermodynamically more stable [1,2,4]triazolo[1,5-*c*]pyrimidines [14,15,16]. This isomerization was first reported by Miller and Rose [17,18] when they treated [1,2,4] triazolo[4,3-*c*]pyrimidine derivatives with an acid, base, or thermally. Based on the above mentioned research results, the goal of this study is to synthesize some novel pyrazolopyrimidine, pyrazolotriazolo[4,3-*c*]pyrimidines and pyrazolotriazolo[1,5-*c*]pyrimidines not only to study their isomerization, but also to obtain new compounds which are expected to possess notable pharmacological applications.

## 2. Results and Discussion

**Scheme 1:**
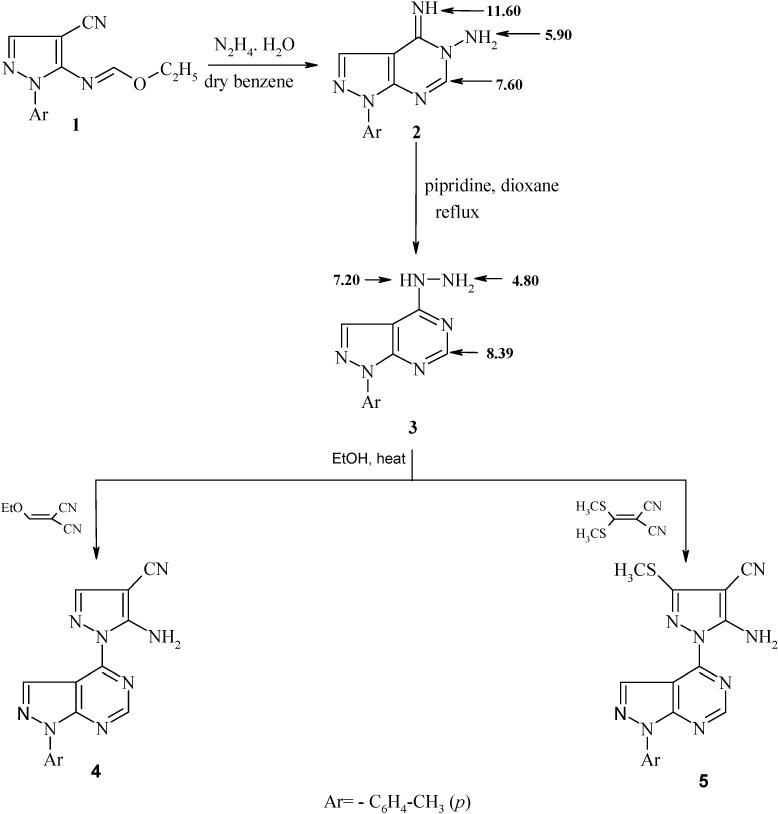
Synthesis of compounds **2**–**5**.

**Scheme 2:**
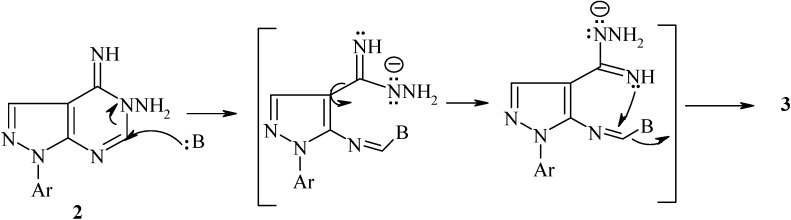
Isomerization of compound **2** to **3**.

Stirring of compound 1 [19], in anhydrous benzene with hydrazine hydrate, afforded 4-imino-1-*p*-tolyl-1,4-dihydro-pyrazolo[3,4-*d*]pyrimidin-5-ylamine (**2**) (Scheme 1). The IR spectrum of the latter compound revealed the absence of the cyano group and the ^1^H-NMR spectrum revealed the absence of the ethoxy group protons and showed signals at 5.90, 11.60 ppm for NH_2_ and imine NH protons, respectively. Moreover, the ^13^C-NMR spectrum of compound **2** revealed a signal at 164.42 ppm for C=NH (C-4). Compound **2** was isomerized to its corresponding more thermodynamically stable 4-hydrazino derivative **3** upon refluxing in dioxane in the presence of a few drops of piperdine. Actually, piperdine acts as a base in this Dimroth-type rearrangement which involves a sequence of ring opening and ring closure reactions (Scheme 2) [15,20,21].

The ^1^H-NMR spectrum of compound **3** revealed signals at 4.80, 7.20 ppm for the NH_2_ and NH, protons, respectively and the ^13^C-NMR spectrum revealed a signal at 168.20 ppm for =C-NHNH_2_ (C-4) (*cf.* Experimental Section). Moreover, the structure of compound **3** was confirmed chemically by refluxing it with ethoxymethylenemalononitrile or bis-(methylthio)methylenemalononitrile to afford the corresponding substituted pyrazole derivatives **4** and **5**, respectively. The IR spectra of the latter compounds showed absorption bands characteristic for NH_2_ and CN groups and the ^1^H-NMR spectra showed signals at δ = 6.79 and 6.85 ppm due to NH_2_ (exchangeable with D_2_O). Also, the MS gave the molecular ion peaks at *m/z* (%) = 316 (23.49) and 362 (33.25) for compounds **4** and **5**, respectively [22,23].

**Scheme 3:**
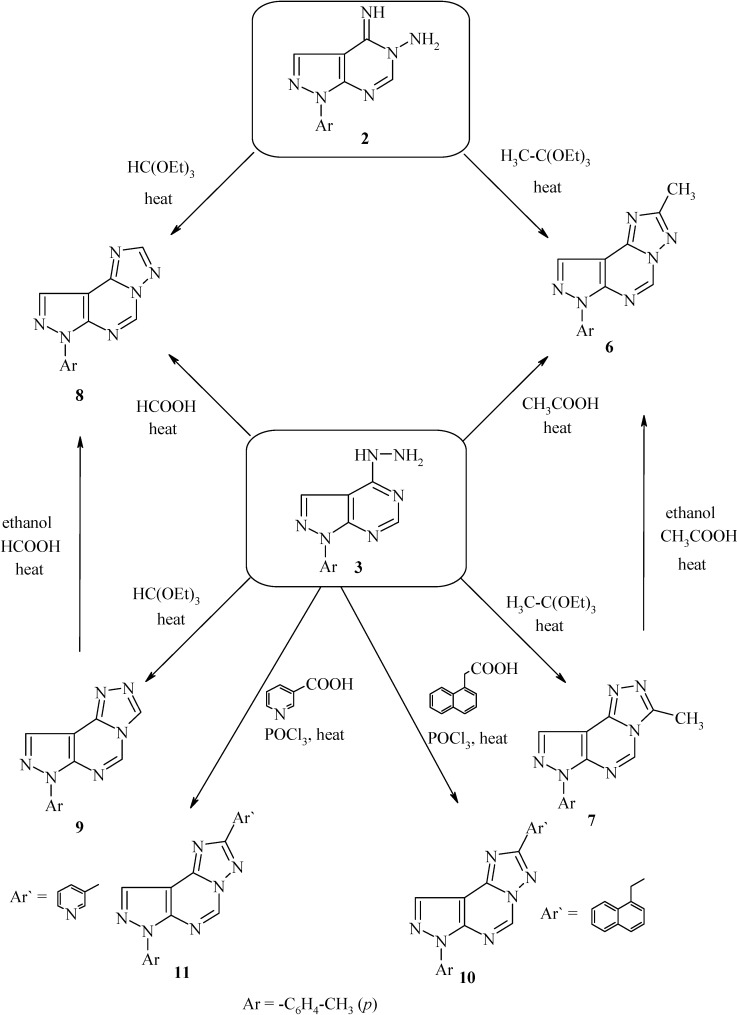
Synthesis of compounds **6**–**11**.

Heating of compounds **2** or **3** with triethyl orthoacetate at its boiling temperature, gave 2-methyl-7-*p*-tolyl-7*H*-pyrazolo[4,3-*e*][1,2,4]triazolo[1,5-*c*]pyrimidine (**6**) or 3-methyl-7-*p*-tolyl-7*H*-pyrazolo[4,3-*e*][1,2,4]triazolo[4,3-*c*]pyrimidine (**7**), respectively (Scheme 3). It was noticed that the two triazolopyrimidine derivatives **6** and **7** showed no appreciable difference in fragmentation pattern under electron impact (*cf.* Experimental Section). However, the ^1^H-NMR spectra of triazolo[4,3-*c*]pyrimidine derivative **7** revealed that the C^3^-CH_3_ and C^5^-H protons appeared at a more downfield location when compared with the C^2^-CH_3_ and C^5^-H protons of the [1,2,4]triazolo[1,5-*c*]pyrimidine derivative **6** (Table 1). These data are in agreement with reported results of related compounds [15,16,17,18,21,22,24] and confirmed that the product obtained from the reaction with hydrazino derivative **3** differ than those obtained from the reaction with imino derivative **2**.

**Table 1:** M.p., ^1^H- and ^13^C-NMR values of triazole and pyrimidine protons of compounds **6**–**9**.

Compd. No.	m.p.	δ ^1^H-NMR					δ ^13^C-NMR				
		C^5^-H	C^3^-H	C^2^-H	C^3^-H_3_	C^2^-CH_3_	C-5	C-3	C-2	C^3^-CH_3_	C^2^-CH_3_
**6**	264–265	9.15	–	–	–	2.55	159.30		160.50		11.60
**7**	275–277	9.35	–	–	2.80	–	162.00	164.27		13.60	
**8**	257–259	9.20	–	9.00	–	–	157.99		158.66		
**9**	290–292	9.40	9.25	–	–	–	160.05	161.90			

However, when compound **3** was heated under reflux in acetic acid, it afforded compound **6**, probably via the intermediacy of its [1,2,4]triazolo[4,3-*c*]pyrimidine isomer **7** which was not isolated in this reaction, but rather underwent a Dimroth-type rearrangement [15,20] under the reaction conditions. To prove this assumption, compound **7** was converted into its corresponding [1,2,4] triazolo[1,5-*c*]pyrimidine derivative **6** by heating in ethanol in the presence of few drops of glacial acetic acid (Scheme 3).

Likewise, when compounds **2** or **3** were refluxed with triethyl orthoformate, it gave 7-*p*-tolyl-7*H*-pyrazolo[4,3-*e*][1,2,4]triazolo[1,5-*c*]pyrimidine (**8**) and 7-*p*-tolyl-7*H*-pyrazolo[4,3-*e*] [1,2,4] triazolo[4,3-*c*]pyrimidine (**9**), respectively (Scheme 3). The ^1^H-NMR spectra of the latter compounds revealed that the C^3^-H and C^5^-H protons of compound **9** appeared more downfield when compared with the C^2^-H and C^5^-H protons of [1,2,4]triazolo[1,5-*c*]pyrimidine derivative **8** (Table 1). Also, when compound **3** was refluxed with formic acid, it afforded compound **8**, probably via the intermediacy of its isomer [1,2,4]triazolo[4,3-*c*]pyrimidine **9**. To prove this assumption, compound **9** was converted into the corresponding [1,2,4] triazolo[1,5-*c*]pyrimidine derivative **8** by heating in ethanol in the presence of few drops of formic acid (Scheme 3). It was noticed that, the NMR spectra and m.p. values for [1,2,4]triazolo[4,3-*c*]pyrimidines **7**, **9** are greater than those of [1,2,4] triazolo[1,5-*c*]pyrimidines **6**, **8** (Table 1).

Moreover, when the hydrazino derivative **3** was refluxed with 1-naphthylacetic acid or nicotinic acid in the presence of phosphorus oxychloride, it afforded the polycyclic 2-naphthyl-3-yl-7-p-tolyl-7*H*-pyrazolo[4,3-*e*][1,2,4]triazolo[1,5-*c*]pyrimidine (**10**) 2-pyridin-3-yl-7-p-tolyl-7*H*-pyrazolo[4,3-*e*][1,2,4]triazolo[1,5-*c*]pyrimidine (**11**), respectively via a Dimroth-type rearrangement of the [1,2,4]triazolo[4,3-*c*]pyrimidine under the acidic reaction conditions.

## 3. Experimental

### General Information

All melting points are uncorrected and were measured using an Electro-Thermal IA 9100 apparatus (Shimadzu, Tokyo, Japan). Infrared spectra were recorded as potassium bromide pellets on a Perkin-Elmer 1650 spectrophotometer (Perkin-Elmer, Norwalk, CT, USA). ^1^H-NMR and ^13^C-NMR spectra were determined on a Jeol-Ex-400-NMR spectrometer (Jeol, Tokyo, Japan) and chemical shifts are expressed as δ values in parts per million using TMS as internal reference. Mass spectra were recorded on a VG 2AM-3F mass spectrometer (Thermo Electron Corporation, Waltham, MA, USA). Microanalyses were obtained using a Mario El Mentar apparatus, and the results were within the accepted range (±0.20) of the calculated values. The reactions were followed and the purity of the compounds was checked by TLC on silica gel-precoated aluminum sheets (Type 60 F254; Merck, Darmstadt, Germany). Compound **1** was prepared according to a reported method (m.p. 82–84 °C; lit*.* [19] m.p. 82–84 °C).

*4-Imino-1-p-tolyl-1,4-dihydropyrazolo[3,4-*d*]pyrimidin-5-ylamine* (**2**): To a solution of compound **1** (2.54 g, 0.01 mol) in anhydrous benzene (30 mL), hydrazine hydrate (99%, 3 mL) was added with stirring for 1 h at room temperature. The obtained product was filtered, dried, and recrystallized from dry dioxane to give compound **2**. Yield 92%; m.p. 163–164 °C. IR (KBr, ν, cm^−1^): 3288, 3262 (NH_2_), and 3199 (NH); ^1^H-NMR (DMSO-*d*_6_, δ ppm): 2.40 (s, 3H, CH_3_), 5.90 (bs, 2H, NH_2_, D_2_O exchangeable), 7.30–7.45 (d, 2H, Ar-H, *J* = 8.5 Hz), 7.50–7.60 (m, 3H, 2Ar-H + C^6^-H), 8.30 (s, 1H, C^3^-H), 11.60 (s, 1H, NH, D_2_O exchangeable). ^13^C-NMR (DMSO-*d*_6_, δ ppm): 20.88 (CH_3_), 103.57 (C-3a), 113.70, 127.82, 130.21, 133.40 (Ar-C), 133.72 (C-3), 154.50 (C-7a), 163.27 (C-6), 164.42 (C-4). MS, *m/z* (%): 240 (M+, 100). Anal. Calcd. for C_12_H_12_N_6_: C, 59.99; H, 5.03; N, 34.98; found: C, 60.06; H, 4.99; N, 34.94.

*(1-p-Tolyl-1H-pyrazolo[3,4-*d*]pyrimidin-4-yl)-hydrazine* (**3**): Compound **2** (1.20 g, 0.005 mol) in anhydrous dioxane (20 mL) containing a few drops of piperidine was refluxed for 6 h. Then the reaction mixture was evaporated under reduced pressure and recrystallized from dioxane to give compound **3**. Yield 83%, m.p. 180–182 °C. IR (KBr, ν, cm^−1^): 3295, 3271 (NH_2_), and 3150 (NH); ^1^H-NMR (DMSO-*d*_6_, δ ppm): 2.45 (s, 3H, CH_3_), 4.80 (s, 2H, NH_2_, D_2_O exchangeable), 7.20 (s, 1H, NH, D_2_O exchangeable), 7.32 (d, 2H, Ar-H, *J* = 8.5 Hz), 7.80 (d, 2H, Ar-H, *J* = 8.5 Hz), 8.31 (s, 1H, C^3^-H), 8.39 (s, 1H, C^6^-H). ^13^C-NMR (DMSO-*d*_6_, δ ppm): 20.90 (CH_3_), 102.75 (C-3a), 113.51, 127.90, 130.35, 133.41 (Ar-C), 133.72 (C-3), 154.50 (C-7a), 158.47 (C-6), 168.20 (C-4). MS, *m/z* (%): 240 (M^+^, 56). Anal. Calcd. for C_12_H_12_N_6_: C, 59.99; H, 5.03; N, 34.98; found: C, 59.93; H, 5.05; N, 35.00.

*General procedure for the synthesis of 5-amino-3-substituted-1-(1-p-tolyl-1H-pyrazolo[3,4-*d*]pyrimidin-4-yl)-1H-pyrazole-4-carbonitriles* **4** *and* **5**: To a solution of compound **3** (1.20 g, 0.005 mol) in ethanol (30 mL), ethoxymethylenemalononitrile, or bis(methylthio)methylenemalononitrile (0.005 mol), was added, respectively. The reaction mixture was heated for 2–3 h. The formed precipitate was filtered off and recrystallized from an appropriate solvent to give compounds **4** and **5**, respectively.

*5-Amino-1-(1-p-tolyl-1H-pyrazolo[3,4-*d*]pyrimidin-4-yl)-1H-pyrazole-4-carbonitrile* (**4**): 2 h, yield: (96%, dioxane); m.p. 222–225 °C. IR (KBr, ν, cm^−1^): 3430, 3300 (NH_2_), 3220 (NH), 2222 (CN). ^1^H-NMR (DMSO-*d*_6_, δ ppm): 2.41 (s, 3H, CH_3_), 6.79 (s, 2H, NH_2_, D_2_O exchangeable), 7.33 (d, 2H, Ar-H, *J* = 8.5 Hz), 8.12 (d, 2H, Ar-H, *J* = 8.5 Hz), 8.33 (s, 1H, C^3^-H), 8.34 (s, 1H, C^3'^-H), 8.50 (s, 1H, C^6'^-H). ^13^C-NMR (DMSO-*d*_6_, δ ppm): 20.64 (CH_3_), 103.06 (C-3'a), 111.04 (C-4), 116.23 (CN), 113.61, 128.01, 130.41, 133.33 (Ar-C), 132.50 (C-3), 134.00 (C-3'), 153.92 (C-5), 154.72 (C-4`), 157.57 (C-6'), 159.33 (C-7'a). MS, *m/z* (%): 316 (M^+^, 23.49). Anal. calcd for C_16_H_12_N_8_: C, 60.75; H, 3.82; N, 35.42. Found: C, 60.80; H, 3.79; N, 35.40.

*5-Amino-3-methylsulfanyl-1-(1-p-tolyl-1H-pyrazolo[3,4-*d*]pyrimidin-4-yl)-1H-pyrazole-4-carbonitrile* (**5**): 3 h, yield: (84%, dioxane); m.p. 209–211 °C. IR (KBr, ν, cm^−1^): 3313, 3299 (NH_2_), 3200 (NH), and 2222 (CN). ^1^H-NMR (DMSO-*d*_6_, δ ppm): 2.40 (s, 3H, CH_3_), 3.76 (s, 3H, SCH_3_), 6.85 (s, 2H, NH_2_, D_2_O exchangeable), 7.32 (d, 2H, Ar-H, *J* = 8.5 Hz), 8.15 (d, 2H, Ar-H, *J* = 8.5 Hz), 8.35 (s, 1H, C^3'^-H), 8.55 (s, 1H, C^6'^-H). ^13^C-NMR (DMSO-*d*_6_, δ ppm): 20.64 (CH_3_), 25.15 (CH_3_), 102.57 (C-3'a), 113.04 (C-4), 115.59 (CN), 113.50, 127.90, 130.30, 133.40 (Ar-C), 133.50 (C-3), 133.70 (C-3'), 153.83 (C-5), 154.43 (C-4'), 157.27 (C-6'), 159.42 (C-7'a). MS, *m/z* (%): 362 (M^+^, 33.25). Anal. calcd for C_17_H_14_N_8_S: C, 56.34; H, 3.89; N, 30.92; S, 8.85. Found: C, 56.26; H, 3.94; N, 30.95; S, 8.80.

### 2-Methyl-7-p-tolyl-7H-pyrazolo[4,3-*e*][1,2,4]triazolo[1,5-*c*]pyrimidine (**6**)

*Method A*: A mixture of compound **2** (1.20 g, 0.005 mol) and triethyl orthoacetate (30 mL) was refluxed for 10 h. The reaction mixture was evaporated till dryness and the remaining solid was recrystallized from dioxane to give compound **6**. Yield 70%, m.p. 264–265 °C. ^1^H-NMR (DMSO-*d*_6_, δ ppm): 2.40 (s, 3H, CH_3_), 2.55 (s, 3H, C^2^-CH_3_), 7.35 (d, 2H, Ar-H, *J* = 8.5 Hz), 8.10 (d, 2H, Ar-H, *J* = 8.5 Hz), 8.50 (s, 1H, C^9^-H), 9.15 (s, 1H, C^5^-H). ^13^C-NMR (DMSO-*d*_6_, δ ppm): 11.6 (C^2^-CH_3_), 20.78 (CH_3_), 107.30 (C-9a), 113.46, 127.16, 130.37, 134.15 (Ar-C), 144.50 (C-9), 147.45 (C-6a), 157.27(C-9b), 159.30 (C-5), 160.50 (C-2). MS *m/z* (%): 264 (M^+^, 18.01). Anal. calcd for C_14_H_12_N_6_: C 63.62, H 4.58, N 31.80. Found: C 63.70, H 4.54, N 31.75.

*Method B*: Compound **3** (1.20 g, 0.005 mol) was heated under reflux temperature in glacial acetic acid (30 mL) for 8 h. The reaction mixture was cooled and poured into water. The formed solid was filtered off, dried and recrystallized from dioxane to give product identical in all aspects with compound **6** obtained before. Yield 77%, m.p. 264–265 °C.

*Method C*: A solution of compound **7** (1.32 g, 0.005 mol) in ethanol (20 mL) containing 3–5 drops of glacial acetic acid was heated under reflux temperature for 30 min. The solvent was removed under reduced pressure leaving a solid product which was recrystallized from dioxane to give compound identical in all aspects with compound **6** obtained before. Yield 62%, m.p. 264–265 °C.

*3-Methyl-7-p-tolyl-7H-pyrazolo[4,3-*e*][1,2,4]triazolo[4,3-*c*]pyrimidine* (**7**): Compound **3** (1.20 g, 0.005 mol) was heated under reflux temperature in triethyl orthoacetate (40 mL) for 5 h. The product which separated on cooling was filtered off, dried and recrystallized from dioxane to give compound 7. Yield 80%, m.p. 275–277 °C. ^1^H-NMR (DMSO-*d*_6_, δ ppm): 2.40 (s, 3H, CH_3_), 2.80 (s, 3H, C^3^-CH_3_), 7.34 (d, 2H, Ar-H, *J* = 8.5 Hz), 8.20 (d, 2H, Ar-H, *J* = 8.5 Hz), 8.50 (s, 1H, C^9^-H), 9.35 (s, 1H, C^5^-H). ^13^C-NMR (DMSO-*d*_6_, δ ppm): 13.6 (C^3^-CH_3_), 20.90 (CH_3_), 107.35 (C-9a), 113.50, 127.17, 130.39, 134.16 (Ar-C), 144.70 (C-9), 147.80 (C-6a), 156.37 (C-9b), 162.00 (C-5), 164.27 (C-3). MS *m/z* (%): 264 (M^+^, 29.98). Anal. calcd for C_14_H_12_N_6_: C 63.62, H 4.58, N 31.80. Found: C 63.55, H 4.60, N 31.82.

### 7-p-Tolyl-7H-pyrazolo[4,3-*e*][1,2,4]triazolo[1,5-*c*]pyrimidine (**8**)

*Method A*: A mixture of compound **2** (1.20 g, 0.005 mol) and triethyl orthoformate (30 mL) was refluxed for 10 h. After cooling, the formed precipitate was filtered off, dried, and recrystallized from dioxane to give compound **8**. Yield 80%, m.p. 257–259 °C. ^1^H-NMR (DMSO-*d*_6_, δ ppm): 2.40 (s, 3H, CH_3_), 7.34 (d, 2H, Ar-H, *J* = 8.5 Hz), 8.20 (d, 2H, Ar-H, *J* = 8.5 Hz), 8.50 (s, 1H, C^9^-H), 9.00 (s, 1H, C^2^-H), 9.20 (s, 1H, C^5^-H). ^13^C-NMR (DMSO-*d*_6_, δ ppm): 20.28 (CH_3_), 106.88 (C-9a), 113.47, 127.14, 130.35, 134.15 (Ar-C), 144.10 (C-9), 147.50 (C-6a), 150.57 (C-9b), 157.99 (C-5), 158.66 (C-2). MS *m/z* (%): 250 (M^+^, 18.01). Anal. calcd for C_13_H_10_N_6_: C, 62.39; H, 4.03; N, 33.58. Found: C, 62.49; H, 4.00; N, 33.55.

*Method B*: Compound **3** (1.20 g, 0.005 mol) was heated under reflux temperature in formic acid (40 mL, 85%) for 10 h. The reaction mixture was cooled and poured into water. The formed solid was filtered off, dried and recrystallized from dioxane to give product identical in all aspects with compound **8** obtained before. Yield 77%, m.p. 257–259 °C.

*Method C*: A solution of compound **9** (1.25 g, 0.005 mol), ethanol (20 mL) containing 3–5 drops of formic acid was heated under reflux temperature for 1 h. The reaction mixture was evaporated to dryness and the remaining solid was recrystallized from dioxane to give compound identical in all aspects with compound **8** obtained before. Yield 50%, m.p. 257–259 °C.

*7-p-Tolyl-7H-pyrazolo[4,3-*e*][1,2,4]triazolo[4,3-*c*]pyrimidine* (**9**): Compound **3** (1.20 g, 0.005 mol) was heated under reflux temperature in triethyl orthoformate (40 mL) for 5 h. The reaction mixture was kept at room temperature overnight, then the solvent was evaporated to dryness and the remaining solid was purified on TLC plate using chloroform: methanol (9:1) as an eluent to separate compound **9** as the major product. Yield 79%, m.p. 290–292 °C. ^1^H-NMR (DMSO-*d*_6_, δ ppm): 2.42 (s, 3H, CH_3_), 7.30 (d, 2H, Ar-H, *J* = 8.5 Hz), 8.15 (d, 2H, Ar-H, *J* = 8.5 Hz), 8.50 (s, 1H, C^9^-H), 9.25 (s, 1H, C^3^-H), 9.40 (s, 1H, C^5^-H). ^13^C-NMR (DMSO-*d*_6_, δ ppm): 20.30 (CH_3_), 106.75 (C-9a), 113.51, 127.16, 130.38, 134.17 (Ar-C), 144.50 (C-9), 147.22 (C-6a), 150.87 (C-9b), 160.05 (C-5), 161.90 (C-3). MS *m/z* (%): 250 (M^+^, 50.74). Anal. calcd for C_13_H_10_N_6_: C, 62.39; H, 4.03; N, 33.58. Found: C, 62.30; H, 4.05; N, 33.60.

*General procedure for the synthesis of 2-substituted-7-p-tolyl-7H-pyrazolo[4,3-e][1,2,4]triazolo[1,5-c]pyrimidines* **10** *and* **11**: A mixture of compound **3** (2.40 g, 0.01 mol) in phosphorus oxychloride (40 mL) and 1-naphthylacetic acid (1.86 g, 1 mmol) or nicotinic acid (1.23 g, 1 mmol) was heated under reflux temperature for 5–6 h. The reaction mixture was poured onto crushed ice and the obtained solid was filtered off, dried, and recrystallized from an appropriate solvent to give compounds **10** and **11** respectively.

*2-Naphthalen-1-ylmethyl-7-p-tolyl-7H-pyrazolo[4,3-*e*][1,2,4]triazolo[1,5-*c*]pyrimidine* (**10**): 5 h, yield: (65%, DMF/H_2_O); m.p. 296–297 °C. ^1^H-NMR (DMSO-*d*_6_, δ ppm): 2.40 (s, 3H, CH_3_), 4.0 (s, 2H, CH_2_), 7.30–8.15 (m, 11H, Ar-H), 8.55 (s, 1H, C^9^-H), 9.20 (s, 1H, C^5^-H). ^13^C-NMR (DMSO-*d*_6_, δ ppm): 20.66 (CH_3_), 35.68 (C^2^-CH_2_), 107.41 (C-9a), 113.46, 123.90, 125.20, 125.40 126.20, 126.40, 126.50, 127.16, 128.30, 130.37, 132.50, 133.40, 134.10, 134.15 (Ar-C), 144.62 (C-9), 147.61 (C-6a), 157.00(C-9b), 158.98 (C-5), 160.40 (C-2). MS *m/z* (%): 390 (M^+^, 33.02). Anal. calcd for C_24_H_18_N_6_: C, 73.83; H, 4.65; N, 21.52. Found: C, 73.76; H, 4.61; N, 21.57.

*2-Pyridin-3-yl-7-p-tolyl-7H-pyrazolo[4,3-*e*][1,2,4]triazolo[1,5-*c*]pyrimidine* (**11**): 6 h, yield: (77%, dioxane); m.p. 288–289 °C. ^1^H-NMR (DMSO-*d*_6_, δ ppm): 2.39 (s, 3H, CH_3_), 7.10–8.15 (m, 8H, Ar-H), 8.50 (s, 1H, C^9^-H), 9.25 (s, 1H, C^5^-H). ^13^C-NMR (DMSO-*d*_6_, δ ppm): 20.54 (CH_3_), 107.42 (C-9a), 113.44, 123.80, 127.20, 130.40, 133.40, 134.16, 134.20, 148.11, 149.45 (Ar-C), 143.92 (C-9), 147.52 (C-6a), 157.22 (C-9b), 157.92 (C-5), 159.80 (C-2). MS *m/z* (%): 327 (M^+^, 33.02). Anal. calcd for C_18_H_13_N_7_: C, 66.04; H, 4.00; N, 29.95. Found: C, 65.96; H, 4.05; N, 29.90.

## 4. Conclusions

The synthesis and structure characterization of new pyrazoles and pyrazolopyrimidines was discussed. Also, structure characterization of pyrazolo[4,3-*e*][1,2,4]triazolo[4,3-*c*]pyrimidine derivatives **7** and **9** and their isomerization to pyrazolo[4,3-*e*][1,2,4]triazolo[1,5-*c*]pyrimidine derivatives **6** and **8**, respectively, under different suitable reaction conditions were reported.
